# Supplementary figures and images for: Trehalose Phosphate Synthase Complex-Mediated Regulation of Trehalose 6-Phosphate Homeostasis Is Critical for Development and Pathogenesis in Magnaporthe oryzae
Source: mSystems. 2021 Oct 5;6(5):e00462-21. doi: 10.1128/mSystems.00462-21 (PMC8547450; doi:10.1128/mSystems.00462-21)

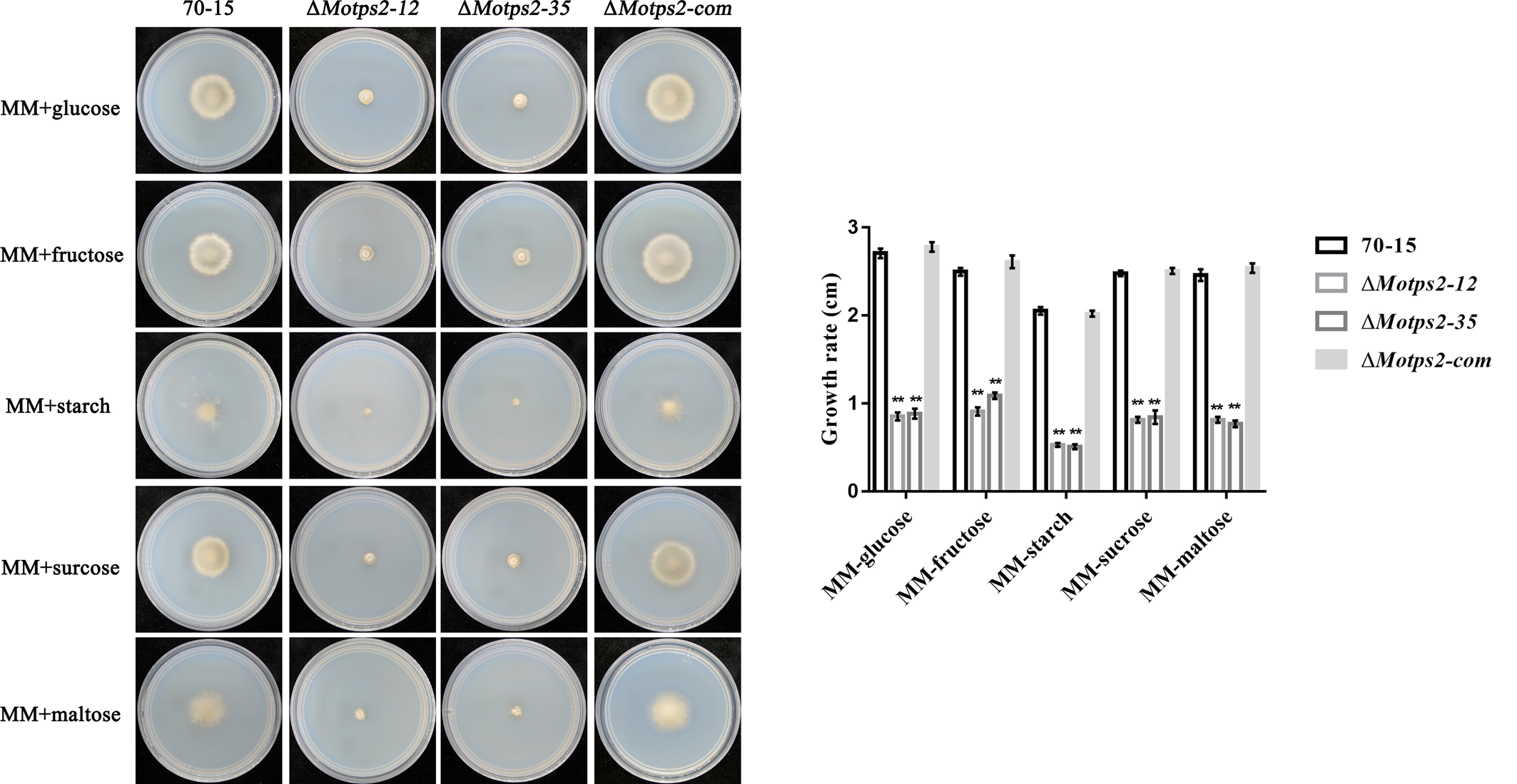

Supplement: FIG S3 [file msystems.00462-21-sf003.tif]

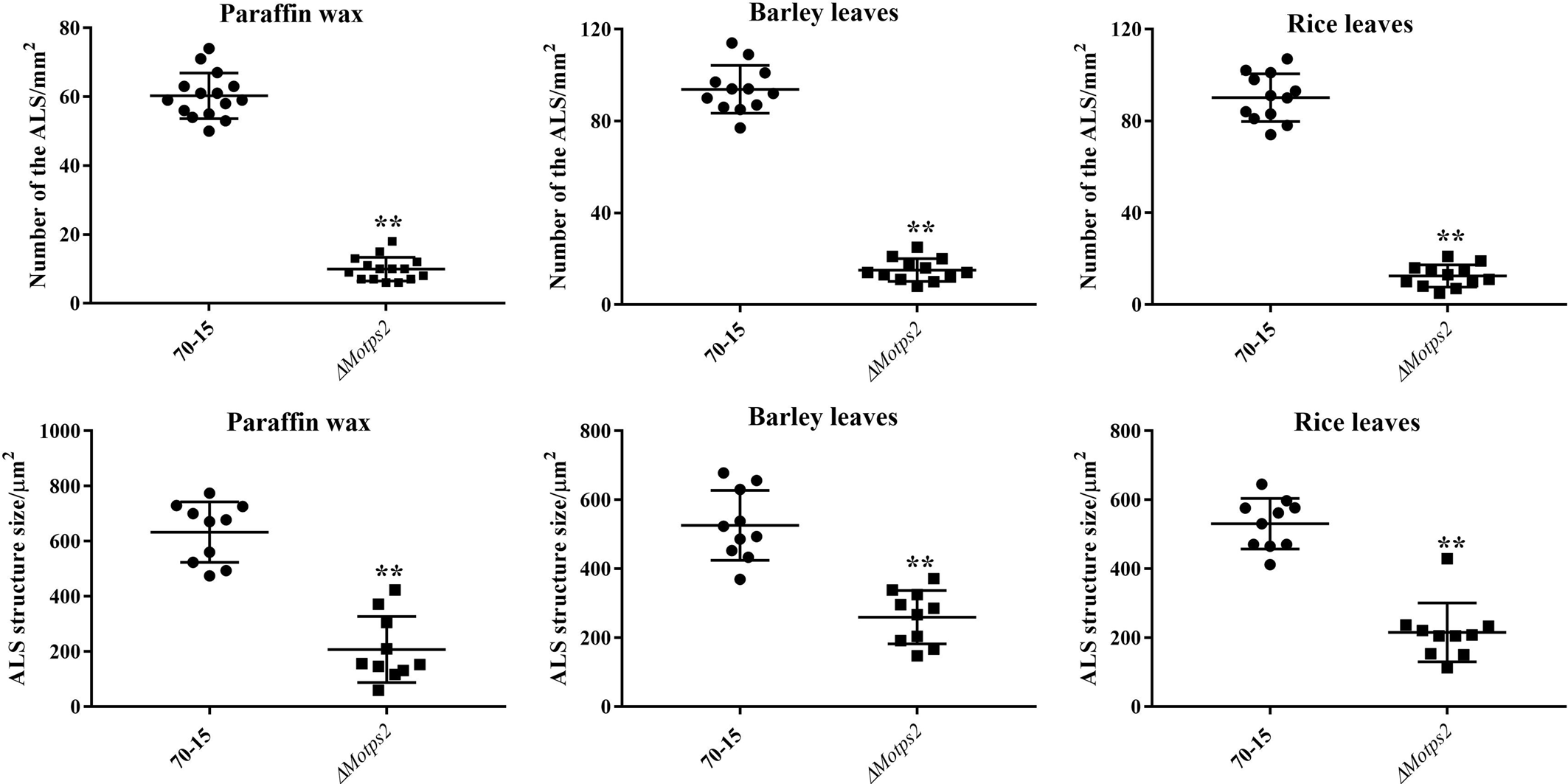

Supplement: FIG S4 [file msystems.00462-21-sf004.tif]

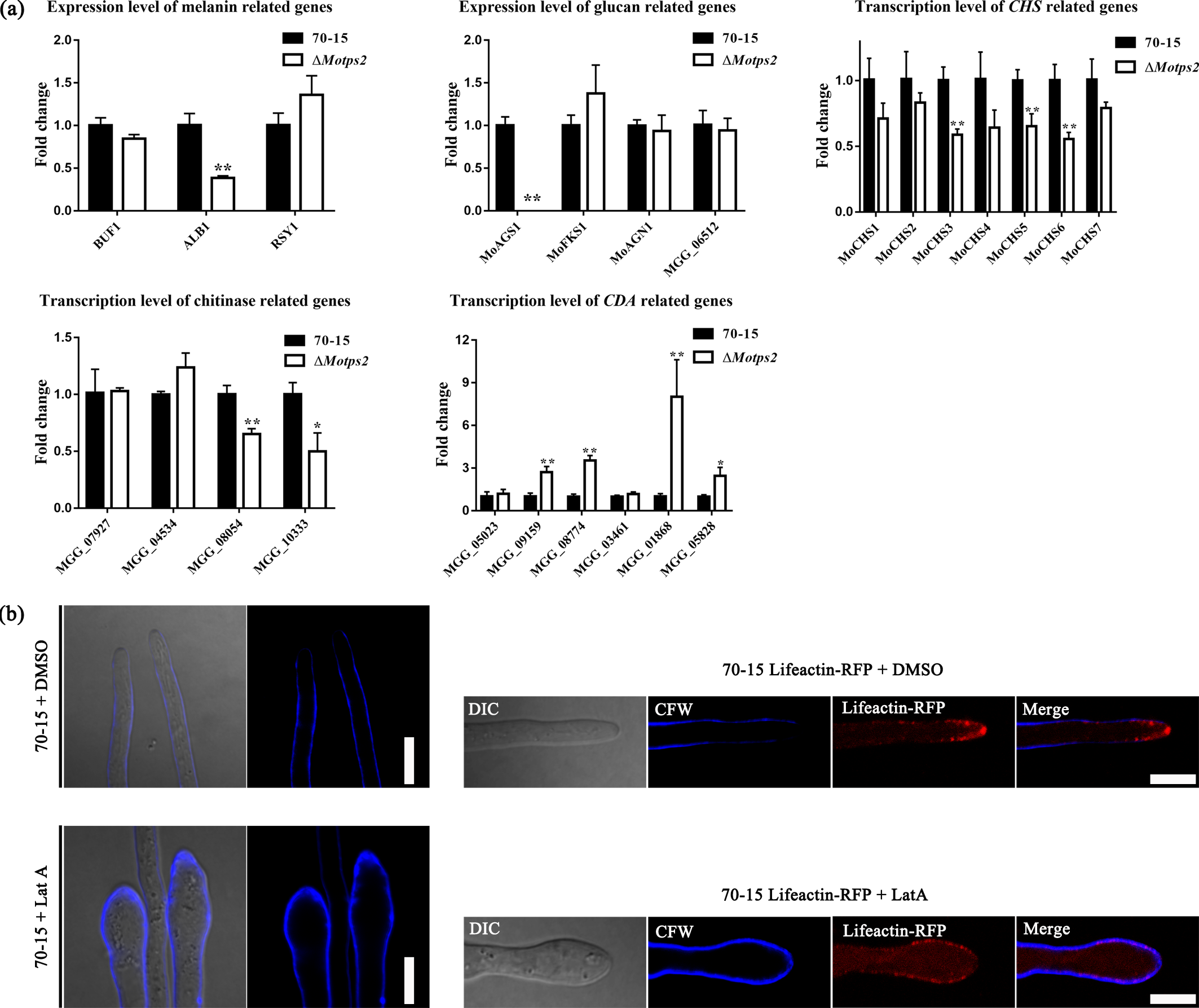

Supplement: FIG S5 [file msystems.00462-21-sf005.tif]

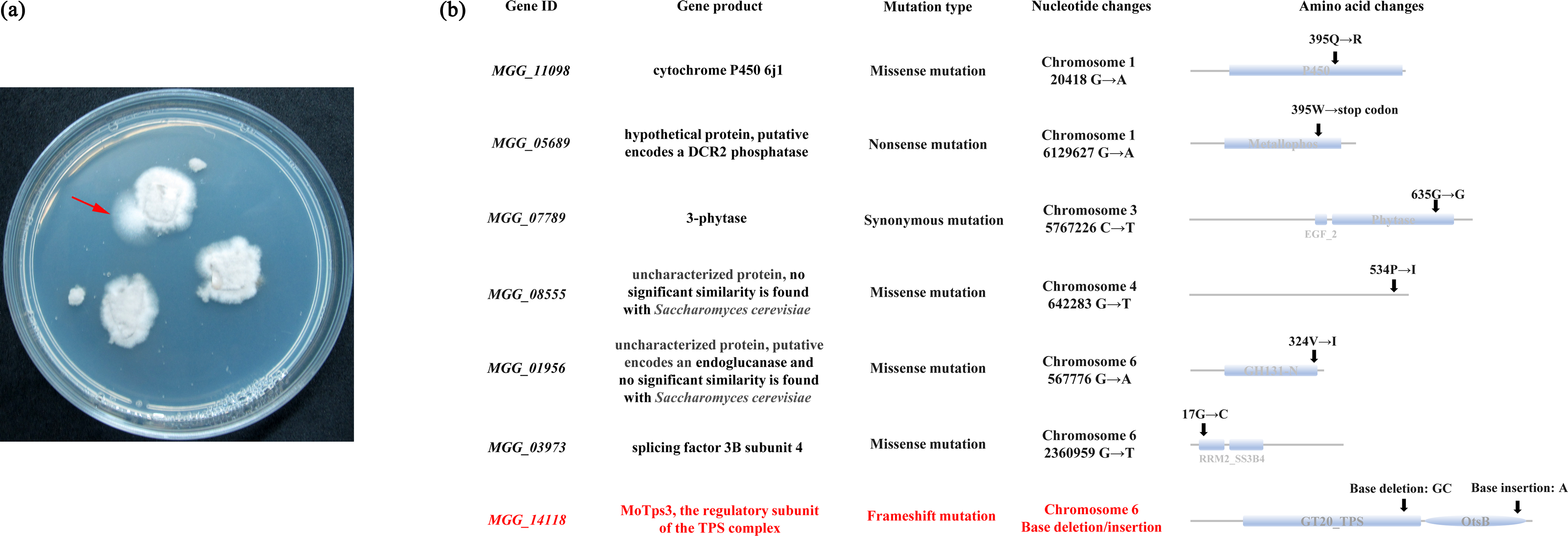

Supplement: FIG S6 [file msystems.00462-21-sf006.tif]

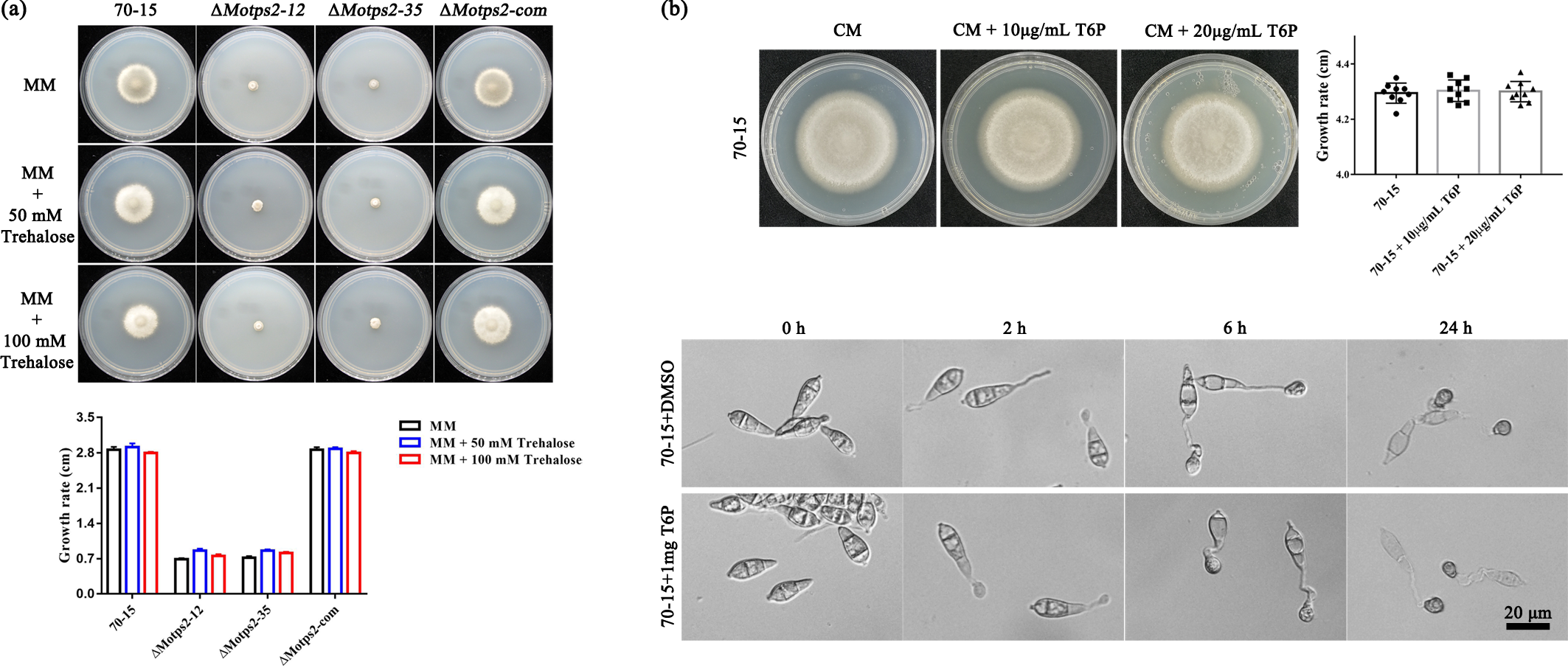

Supplement: FIG S7 [file msystems.00462-21-sf007.tif]
