# Supplementary material for: Trehalose Phosphate Synthase Complex-Mediated Regulation of Trehalose 6-Phosphate Homeostasis Is Critical for Development and Pathogenesis in Magnaporthe oryzae
Source: mSystems. 2021 Oct 5;6(5):e00462-21. doi: 10.1128/mSystems.00462-21 (PMC8547450; doi:10.1128/mSystems.00462-21)
Supplement: TABLE S3 [file msystems.00462-21-st003.docx]

**Table S3 Metabolites differences among the indicated strains**

| Primary  metabolites | Δ*Motps2*  vs 70-15 | Δ*Motps2-m*  vs 70-15 | Δ*Motps2-* Δ*Motps1*  vs 70-15 | Δ*Motps2- TreC*  vs 70-15 |
| --- | --- | --- | --- | --- |
| pyruvic acid | **-0.314** (*0.0006*) | **-0.234** (*0.031*) | **-0.250** *(0.014*) | **-0.189** (*0.022*) |
| lactic Acid | **-0.400** (*0.006*) | **-0.246** (*0.014*) | **0.036** (*0.791*) | **-0.004** (*0.886)* |
| succinic acid | **-0.578** (*0.027*) | **-0.239** (*0.207*) | **0.009** (*0.6974*) | **-0.049** (*0.932*) |
| fumaric acid | **-0.391** (*0.006*) | **-0.097** (*0.952*) | **-0.098** (*0.296*) | **0.108** (*0.594*) |
| malic acid | **-0.383** *(0.029*) | **-0.134** *(0.248*) | **-0.141** (*0.162*) | **-0.069** (*0.408*) |
| 4-aminobutyric acid | **0.094** (*0.363*) | **0.078** (*0.732*) | **0.232** (*0.292)* | **0.884** (*0.007*) |
| α-ketoglutaric acid | **-0.660** (*0.001*) | **-0.176** (*0.087*) | **-0.091** (*0.116*) | **-0.410** (*0.005*) |
| citric acid | **-0.813** (*0.045*) | **-0.255** (*0.308*) | **-0.404** (*0.152*) | **-0.807** (*0.045*) |
| fructose | **0.233** (*0.241*) | **0.339** (*0.183*) | **-0.630** (*0.023*) | **-0.495** (*0.025*) |
| glucose | **0.172** (*0.252*) | **-0.269** (*0.133*) | **-0.727** (*0.041*) | **-0.724** (*0.031*) |
| glucose-6-phosphate | **-0.754** (*0.002*) | **-0.430** (*0.068*) | **0.317** (*0.339*) | **0.524** (*0.105*) |
| alanine | **-0.372** (*0.023*) | **-0.362** (*0.019*) | **-0.310** (*0.026*) | **-0.112** (*0.163*) |
| valine | **-0.387** (*0.002*) | **-0.325** (*0.078*) | **-0.133** (*0.055*) | **-0.002** (*0.971*) |
| leucine | **-0.310** (*0.041*) | **-0.214** (*0.233*) | **-0.221** (*0.072*) | **-0.206** (*0.071*) |
| isoleucine | **-0.071** (*0.071*) | **-0.083** (*0.479*) | **1.054** (*0.004*) | **0.072** (*0.098*) |
| proline | **-0.082** (*0.534*) | **-0.452** (*0.068*) | **-0.390** (*0.071*) | **-0.261** (*0.140*) |
| glycine | **-0.110** (*0.489*) | **0.045** (*0.843*) | **-0.299** (*0.137*) | **-0.208** (*0.212*) |
| serine | **-0.188** (*0.194*) | **-0.094** (*0.585*) | **-0.157** (*0.230*) | **-0.059** (*0.647*) |
| L-threonine | **-0.186** (*0.111*) | **-0.086** (*0.578*) | **-0.370** (*0.036*) | **-0.105** (*0.251*) |
| methionine | **-0.645** (*0.011*) | **0.113** (*0.333*) | **-0.356** (*0.061*) | **-0.228** (*0.087*) |
| glutamic acid | **-0.625** (*0.006*) | **0.226** (*0.046*) | **-0.005** (*0.961*) | **-0.193** (*0.077*) |
| phenylalanine | **-0.394** (*0.046*) | **-0.053** (*0.602*) | **-0.480** (*0.039*) | **-0.271** (*0.088*) |
| asparagine | **-0.374** (*0.027*) | **0.124** (*0.695*) | **-0.188** (*0.235)* | **-0.099** (*0.471*) |
| glutamine | **-0.757** (*0.002*) | **0.282** (*0.136*) | **-0.478** (*0.029*) | **0.439** (*0.033*) |
| lysine | **-0.716** (*0.042*) | **-0.008** (*0.681*) | **-0.343** (*0.222*) | **-0.237** (*0.257*) |
| tyrosine | **-0.344** (*0.009*) | **0.271** (*0.034*) | **0.217** (*0.065*) | **0.259** (*0.028*) |
| aspartic acid | **-0.530** (*0.002*) | **-0.108** (*0.045*) | **-0.716** (*0.006*) | **-0.213** (*0.117*) |

1 Data in bold and italic represent fold change and *P* value, respectively.

2 Positive and negative signs represent increase or decrease in metabolites content as compare to wild type 70-15
