# Supplementary material for: Trehalose Phosphate Synthase Complex-Mediated Regulation of Trehalose 6-Phosphate Homeostasis Is Critical for Development and Pathogenesis in Magnaporthe oryzae
Source: mSystems. 2021 Oct 5;6(5):e00462-21. doi: 10.1128/mSystems.00462-21 (PMC8547450; doi:10.1128/mSystems.00462-21)
Supplement: TABLE S1 [file msystems.00462-21-st001.docx]

**Table S1 Strains used in this study**

| Strain | Description | References |
| --- | --- | --- |
| 70-15 | Wild type | [1] |
| Δ*Motps2* | *MoTPS2* deletion mutant of 70-15 | This study |
| Δ*Motps1* | *MoTPS1* deletion mutant of 70-15 | This study |
| Δ*Motps2-*Δ*Motps1* | *MoTPS1* deletion mutant of Δ*Motps2* | This study |
| Δ*Motps2-m* | Spontaneous mutation of Δ*Motps2* mutant | This study |
| Δ*Motps2-*Δ*Motps3* | *MoTPS3* deletion mutant of Δ*Motps2* | This study |
| 70-15-TreC | TreC-GFP transformant of 70-15 | This study |
| Δ*Motps2-TreC* | TreC-GFP transformant of Δ*Motps2* mutant | This study |
| Δ*Motps2-com* | MoTps2-GFP transformant of Δ*Motps2* mutant | This study |
| 70-15-Lifeactin-RFP | Lifeactin-RFP transformant of 70-15 | This study |
| Δ*Motps2*-Lifeactin-RFP | Lifeactin-RFP transformant of Δ*Motps2* mutant | This study |
| Δ*Motps2*-m-Lifeactin-RFP | Lifeactin-RFP transformant of Δ*Motps2-m* mutant | This study |
| 70-15-MoChs1-GFP | MoChs1-GFP transformant of 70-15 | This study |
| Δ*Motps2*-MoChs1-GFP | MoChs1-GFP transformant of Δ*Motps2* mutant | This study |
| Δ*Motps2-m*-MoChs1-GFP | MoChs1-GFP transformant of Δ*Motps2-m* mutant | This study |
| 70-15-MoChs3-GFP | MoChs3-GFP transformant of 70-15 | This study |
| Δ*Motps2*-MoChs3-GFP | MoChs3-GFP transformant of Δ*Motps2* mutant | This study |
| Δ*Motps2*-m-MoChs3-GFP | MoChs3-GFP transformant of Δ*Motps2-m* mutant | This study |
| MoTps2-NYFP+ MoTps1-CYFP | MoTps2-NYFP and MoTps1-CYFP co-transformant of 70-15 | This study |
| MoTps3-NYFP+ MoTps1-CYFP | MoTps3-NYFP and MoTps1-CYFP co-transformant of 70-15 | This study |
| MoTps3-NYFP + MoTps2-CYFP | MoTps3-NYFP and MoTps2-CYFP co-transformant of 70-15 | This study |
| MoTps3-NYFP + pCX62-CYFP | MoTps3-NYFP and pCX62-CYFP co-transformant of 70-15 | This study |
| MoTps2-NYFP + pCX62-CYFP | MoTps2-NYFP and pCX62-CYFP co-transformant of 70-15 | This study |
| MoTps2-CYFP + pKNT-NYFP | MoTps2-CYFP and pKNT-NYFP co-transformant of 70-15 | This study |
| MoTps1-CYFP + pKNT-NYFP | MoTps1-CYFP and pKNT-NYFP co-transformant of 70-15 | This study |
| WT-MoSpa2-GFP | MoSpa2-GFP transformant of 70-15 | This study |
| Δ*Motps2*-MoSps2-GFP | MoSpa2-GFP transformant of Δ*Motps2* mutant | This study |
| Δ*Motps2-m*-MoSps2-GFP | MoSpa2-GFP transformant of Δ*Motps2-m* mutant | This study |

1. Dean RA, Talbot NJ, Ebbole DJ, Farman ML, Mitchell TK, Orbach MJ, et al. The genome sequence of the rice blast fungus *Magnaporthe grisea*. Nature. 2005;434(7036):980-6. Epub 2005/04/23. doi: 10.1038/nature03449. PMID: 15846337
