# Supplementary material for: Trehalose Phosphate Synthase Complex-Mediated Regulation of Trehalose 6-Phosphate Homeostasis Is Critical for Development and Pathogenesis in Magnaporthe oryzae
Source: mSystems. 2021 Oct 5;6(5):e00462-21. doi: 10.1128/mSystems.00462-21 (PMC8547450; doi:10.1128/mSystems.00462-21)
Supplement: TABLE S2 [file msystems.00462-21-st002.docx]

**Table S2 Primers used in this study**

| Primers | Sequence (5'→3') | Application |
| --- | --- | --- |
| MoTps2 AF | TACCGTAAACCCGTCAAGACCAT | Construction of Δ*Motps2,* Δ*Motps1,* Δ*Motps2-*Δ*Motps1* and Δ*Motps2-*Δ*Motps3* deletion mutants |
| MoTps2 AR | ttgacctccactagctccagccaagccCTCCCAACCTAGTATTCAGCACAGA |  |
| MoTps2 BF | gaatagagtagatgccgaccgcgggttATCTTTCGTCTTTGAACTGTTG |  |
| MoTps2 BR | CATGTATCGTGTTCGCTTAAC |  |
| MoTps2 OF | AACCGAGGAAGGCATTACGCT |  |
| MoTps2 OR | GCACGCTCGTGATAAGTCCAAT |  |
| MoTps2 UA | CGATGTTATTCACCGCTCCTCT |  |
| Tps1AF | CCCTCAACTTGCTTATCG |  |
| Tps1HAR | ttgacctccactagctccagccaagccAACCTTTCAGGCTGTGCT |  |
| Tps1GAR | tggaaattgtaagcgttaatctagaAACCTTTCAGGCTGTGCT |  |
| Tps1OF | CCGACCACAACACCAAGA |  |
| Tps1OR | AGGAAATGCCGTGCGTAA |  |
| Tps1HBF | gaatagagtagatgccgaccgcgggttGAAAGTAGGCGTAGCAGG |  |
| Tps1BR | GGCAGCGACAAGAACATC |  |
| TPS1GBF | ttcttgacgagttcttctgaGAAAGTAGGCGTAGCAGG |  |
| Tps1UA | GCTACCACTACGCCACAA |  |
| Tps3 AF | TTTTGAACTTGGCTGGTG |  |
| TPS3 GAR | tggaaattgtaagcgttaatctagaCGATTTGTCGTAGCGTCT |  |
| TPS3 GBF | ttcttgacgagttcttctgaTCAACAGCCAAATCACCC |  |
| TPS3 BR | TCCCGAACGCCAAACTCT |  |
| TPS3 OF | GAACGCCACCTCTACTAATCC |  |
| TPS3 OR | GTCACCCACGACCATCAAA |  |
| TPS3 UA | TGTGGCTCTGTCTGGATT |  |
| MoTps1 GF | agggaacaaaagctgggtaccGTAGATGCGAATGTTTGCAC | Subcellular localization examination |
| MoTps1 GR | gcccttgctcaccataagcttGTTTCCCTCCGTCTTGTTGT |  |
| MoTps2 GF | agggaacaaaagctgggtaccCGTCAAGACCATCAACATCC |  |
| MoTps2 GR | gcccttgctcaccataagcttCTCAATATCGGGCACCCTAC |  |
| MoTps3 GF | agggaacaaaagctgggtaccTGTTCATTCTACTGGCAGTG |  |
| MoTps3 GR | gcccttgctcaccataagcttCGAGAGCGCAGCCAGCTTCT |  |
| MoChs1 GF | agggaacaaaagctgggtaccCGTTGGCTTTGTTGTCCCTT |  |
| MoChs1 GR | gcccttgctcaccataagcttGCGACGGGCAATGCAGCACA |  |
| MoChs3 GF | agggaacaaaagctgggtaccCGGAACAATGAATGTACGAG |  |
| MoChs3 GR | gcccttgctcaccataagcttAACGCCCCTAAACATACGAA |  |
| TreC OEF | aacccaatcttcaaactcgagATGACTCATCTTCCCCACTG |  |
| TreC OER | gcccttgctcaccataagcttCTTCTGTAACCACCAGACAG |  |
| CHS1 QF | AGTGCGTTCGGATACGTTAG | qRT-PCR analysis  qRT-PCR analysis |
| CHS1 QR | TGGTCACCGTGGAAGTATTG |  |
| CHS2 QF | CGGTCCTCTTAGCCAGTATTTC |  |
| CHS2 QR | TACGATCCTCAGCCAGATACA |  |
| CHS3 QF | CAACGAGGACGAGGTTCTTT |  |
| CHS3 QR | TCTTCTTCCATGCCTCCTTTC |  |
| CHS4 QF | GAAGAGCTACGCGACAAGAA |  |
| CHS4 QR | CTCGAAGCATTCAGCGATTTG |  |
| CHS5 QF | GACTCTTGTTGGCGGTCTTTA |  |
| CHS5 QR | CATCCCGGATACGAGCTAATTG |  |
| CHS6 QF | CTTCGCTGGTGAGGTTGAATA |  |
| CHS6 QR | GACCGAAGAGACGAGCAATAAA |  |
| CHS7 QF | CTCGTGACTCATTCGACAGTT |  |
| CHS7 QR | CGATCCTCGTCACCCATTATAC |  |
| MGG_07927 QF | ATCACGACTAACCTGCTTTCTT |  |
| MGG_07927 QR | TGTTTGGCTGTGGTGTAGAG |  |
| MGG_04534 QF | CATTTGCGCACACCAATCTC |  |
| MGG_04534 QR | TAGAAGCCCAATCCCAAGTTC |  |
| MGG_08054 QF | CCCACGTTGTGTCATGTATCT |  |
| MGG_08054 QR | GCCATTCCTTGCGACTATCT |  |
| MGG_10333 QF | CACAAGAGGGCCTTGGTTATTA |  |
| MGG_10333 QR | CCCACATTCGCAAAGTTGATG |  |
| CD1 QF | CATGCAGCGATAAGCATTGG |  |
| CD1 QR | GCACTGACCAAACTGAGACT |  |
| CD2 QF | AGATGGTCAAGAACGAGATGG |  |
| CD2 QR | GTCGAAGTAGGTGATGTGGTAG |  |
| CD3 QF | TGATCGCTCTGACGTTTGAC |  |
| CD3 QR | TCTTGCCGTTGAGGAAGAAG |  |
| CD4 QF | GATGAAGCTCCTCCAGGTACTA |  |
| CD4 QR | CCCTCCGGAAGATATCGAGAG |  |
| CD5 QF | GCCAACTTCAGCATCAGAAATG |  |
| CD5 QR | GTCATCTCCAGGGTGTTTGT |  |
| CD6 QF | GTACGACTGCAACGACTTCA |  |
| CD6 QR | CTCCTCGTACAGCACATCAAA |  |
| MoTPS1 QF | GAGCTCATGCTCACGTATCTT |  |
| MoTPS1 QR | AACTTCTCCGGGTCAATTCC |  |
| MoTPS2 QF | ACTACAAGCCGGGTGATATTG |  |
| MoTPS2 QR | ACGGAGAGTGGAGGAAGAA |  |
| MoTPS3 QF | GCAACTTGAAGACCGCTTTG |  |
| MoTPS3 QR | GAGATGCTTGCCCTCGTATT |  |
| MoTps1 BDF | ctgatctcagaggaggacctgcatATGGGCAGCGTTGAAGACGA | Yeast two-hybrid assay |
| MoTps1 BDR | cgctgcaggtcgacggatccccgggaaTCAGTTTCCCTCCGTCTTGT |  |
| MoTps2 ADF | gacgtaccagattacgctcatATGTCGCAGGATGCAGCGTC |  |
| MoTps2 ADR | tatcgatgcccacccgggtggaaCTACTCAATATCGGGCACCC |  |
| MoTps2 BDF | ctgatctcagaggaggacctgcatATGTCGCAGGATGCAGCGTC |  |
| MoTps2 BDR | cgctgcaggtcgacggatccccgggaaCTACTCAATATCGGGCACCC |  |
| MoTps3 ADF | gacgtaccagattacgctcatATGACAGTTTTTATTGCTTC |  |
| MoTps3 ADR | tatcgatgcccacccgggtggaaTCACGAGAGCGCAGCCAGCT |  |
| Tps1 CYFP-F | agggaacaaaagctgggtaccGTAGATGCGAATGTTTGCAC | BiFC assay  BiFC assay |
| Tps1 CYFP-R | cttgcaggccgggcgaagcttGTTTCCCTCCGTCTTGTTGT |  |
| Tps3 NYFP-F | agggaacaaaagctgggtaccTGTTCATTCTACTGGCAGTG |  |
| Tps3 NYFP-R | cgtggcgatggagcgaagcttCGAGAGCGCAGCCAGCTTCT |  |
| Tps2 NYFP-F | agggaacaaaagctgggtaccCGTCAAGACCATCAACATCC |  |
| Tps2 NYFP-R | cgtggcgatggagcgaagcttCTCAATATCGGGCACCCTAC |  |
| Tps2 CYFP-F | agggaacaaaagctgggtaccCGTCAAGACCATCAACATC |  |
| Tps2 CYFP-R | cttgcaggccgggcgaagcttCTCAATATCGGGCACCCTAC |  |
